# Rapamycin potentiates cytotoxicity by docetaxel possibly through downregulation of Survivin in lung cancer cells

**DOI:** 10.1186/1756-9966-30-28

**Published:** 2011-03-10

**Authors:** Huiyan Niu, Jiahe Wang, Hui Li, Ping He

**Affiliations:** 1 Department of Geriatrics, Shengjing Hospital, China Medical University, Shenyang 110004, China

## Abstract

**Background:**

To elucidate whether rapamycin, the inhibitor of mTOR (mammalian target of rapamycin), can potentiate the cytotoxic effect of docetaxel in lung cancer cells and to probe the mechanism underlying such enhancement.

**Methods:**

Lung cancer cells were treated with docetaxel and rapamycin. The effect on the proliferation of lung cancer cells was evaluated using the MTT method, and cell apoptosis was measured by flow cytometry. Protein expression and level of phosphorylation were assayed using Western Blot method.

**Results:**

Co-treatment of rapamycin and docetaxel was found to favorably enhance the cytotoxic effect of docetaxel in four lung cancer cell lines. This tumoricidal boost is associated with a reduction in the expression and phosphorylation levels of Survivin and ERK1/2, respectively.

**Conclusion:**

The combined application of mTOR inhibitor and docetaxel led to a greater degree of cancer cell killing than that by either compound used alone. Therefore, this combination warrants further investigation in its suitability of serving as a novel therapeutic scheme for treating advanced and recurrent lung cancer patients.

## Background

Despite recent advancement in the multidisciplinary treatment of cancer, the prognosis for lung cancer remains poor in more advanced stages and recurrent cases. According to World Health Organization, lung cancer ranks at the top in cancer-related mortalities in humans, killing more than one million people each year.

Mammalian target of rapamycin (mTOR), a serine/threonine protein kinase of 289 kDa, is critically involved in cellular signal transduction mediated by phosphatidylinositol 3 kinase (PI3K)[1]. The activation of mTOR results in changes in multiple cellular processes, e.g., catabolism, anabolism, proliferation, growth and apoptosis[2,3]. Although mTOR is expressed in virtually all mammalian cells, it is believed to play a particularly important role in cancer cells[4-7]. Recent reports have suggested that PI3K/Akt/mTOR pathway is often activated in various forms of lung cancer and that this pathway is considered to be important for cancer cells' survival, proliferation, angiogenesis and resistance to chemotherapy. This pathway can, therefore, be regarded as an attractive target of molecular targeting therapy[8].

Docetaxel (DTX) is one of the most effective chemotherapeutic agents used in the treatment of advanced non-small cell lung cancer (NSCLC). Its anticancer effect is believed to be associated with its ability to induce the polymerization of tubulin, which in turn leads to mitotic arrest. In clinical applications involving lung cancer patients, docetaxel could be either taken together with a platinum compound such as cistaplatin for the first-line treatment or used alone in the second-line treatment of advance stages of NSCLC[9-11]. However, it appears that cancer cells can adapt to become resistant to docetaxel. This currently poses a major clinical problem, because it reduces markedly the effectiveness of docetaxel in the treatment of cancers.

Docetaxel has also been the standard of care for other solid tumors such as breast, head and neck, ovarian and prostate cancers, etc. It was reported that the activation of the PI3K/Akt/mTOR signalling pathway can cause ovarian cancer cells to develop resistance to taxane during the course of the therapy[12]. However, a combination treatment using specific PI3K inhibitor and paclitaxel seemed more effective than using paclitaxel alone not only in the reduction of tumor growth, but also in minimizing side effects[12].

Rapamycin and related compounds are molecular targeting agents that specifically inhibit the mammalian target of rapamycin (mTOR). Originally intended for use in transplantation procedures to prevent organ or graft rejection, rapamycin has recently become of significant interest as a potential anti-cancer drug. It has been reported that rapamycin can exert antitumor activity with cytostatic activities such as G1 phase arrest and that it can exhibit anti-angiogenesis properties[13,14]. Rapamycin was also demonstrated to have synergistic cytotoxic effect in conjunction with other chemotherapeutic agents on several cancer cell types[15-19]. Several rapamycin analogues have been synthesized and put under evaluation in phase Ⅰ/Ⅱ clinical trials, showing a promising antitumor effect in several types of refractory or advanced tumors. This evidence prompted us to examine whether the administration of rapamycin will result in some beneficial modulation of the cancer killing properties of docetaxel in lung cancer cells[20,21].

To the best of our knowledge, the effect of including rapamycin in combination therapies intended to treat advanced stage lung cancer has not been reported in the literature. This prompted us to examine whether juxtaposed administration of rapamycin will result in some beneficial modulation of the cancer killing properties of docetaxel in lung cancer cells. Our results showed that rapamycin can sensitize lung cancer cells for more effective killing by docetaxel and suggested that such enhancement may involve down-regulation of the expression of Survivin and the inactivation of ERK signalling.

## Materials and methods

### Therapeutic compounds and reagents

Lung cancer cell lines A549, SPC-A-1, 95D and NCI-H446 were purchased from Shanghai Institue of Biochemistry and Cell Biology, Chinese Academy of Sciences. Rapamycin, DMSO and MTT were purchased from Sigma (St Louis, MO, USA). Docetaxel was purchased from Shanghai Sanwei Pharmaceutical Company (Shanghai, China). Annexin V-FITC apoptosis detection kit was from Jingmei Biotech (Shenzhen, China). RPMI tissue culture medium and fetal bovine serum (FBS) were purchased from GIBCO (USA). Anti-Survivin, anti-caspase-3, anti-ERK1/2, anti-p-ERK1/2, anti-GAPDH and HRP-conjugated secondary antibodies were purchased from Santa Cruz Biotechnology (CA, USA). Chemiluminescence (ECL) reagent kit was purchased from Pierce Biotechnology (Rockford, IL, USA).

### Cell culture

A549, SPC-A-1, 95D and NCI-H446 cells were cultured in RPMI-1640 medium containing 10% fetal bovine serum, 100 IU/ml penicillin and 100 μg/ml streptomycin. The cells were grown in a humidified incubator at 37°C and in an atmosphere of 5% CO_2 _in air. Cells were grown on sterile tissue culture *petri *dishes and passaged once every 2 to 3 days.

### MTT cell viability assay

Cell were seeded in a 96-well plate at a density of 1 × 10^6^/ml and cultured in medium for 24 h. Cell viability was determined using the conversion of MTT to formazan via mitochondrial oxidation. Various treatments of cells included the addition of rapamycin (12.5 nM, 25 nM, 50 nM, 100 nM), docetaxel (1 nM, 10 nM, 50 nM, 100 nM) and the combination of docetaxel and 20 nM rapamycin for 24 h. Cells in the control group were treated with only the DMSO solution used to dilute rapamycin. MTT solution was then added to each well at a final concentration of 1 mg/ml per well and the plates were incubated at 37°C for another 4 h. After incubation, 150 μl DMSO was added to each well to dissolve the formazan formed and the absorbance was read at 490 nm using a spectrophotometer.

### Flow cytometry apoptosis assay

Cellular apoptosis was determined using the Annexin V-FITC and propidium iodide (PI) double staining kit according to the manufacturer's protocol. Briefly, 95D cells were seeded in six-well plates and allowed to attach overnight; they were then treated with 20 nM rapamycin (Rapa), 10 nM docetaxel (DTX) alone or a combination (20 nM Rapa + 10 nM DTX). After 48 h, cells were harvested, washed twice with cold PBS, resuspended in 250 μl of binding buffer, and stained with staining solution containing Annexin V/FITC and PI. After incubation in the dark for 30 min, cells were analyzed by FACSCalibur flow cytometry (BD Biosciences).

### Western blot

Western Blotting was performed using standard techniques as previously described[22]. Briefly, cells were washed twice with PBS buffer and lysed in RIPA lysis buffer (50 mM Tris-Cl pH 7.4, 150 mM NaCl, 0.5% sodium deoxycholate, 1% NP-40, 0.1% SDS, 1 mM EDTA, 100 mM NaF, 1 mM Na_3_VO_4_, 1 mM PMSF, and 2 μg/ml aprotinin) on ice. 50 μg total proteins were subjected to sodium dodecyl sulfate-polyacrylamide gel electrophoresis (SDS-PAGE) and transferred to polyvinylidene difluoride (PVDF) membranes. PVDF membranes were blocked with 5% nonfat milk in TBST (10 mM Tris, pH 7.4, 150 mM NaCl and 0.1% Tween-20) at room temperature for 2 h and incubated with the indicated primary antibodies at 4°C overnight with gentle rocking. After washing with TBST, the membranes were reacted with appropriate horseradish peroxidase (HRP)-conjugated secondary antibodies for 1 h at room temperature. After extensive washing with TBST, the presence of proteins was visualized by the enhanced chemiluminescence (ECL) detection kit in accordance with the manufacture's recommendation.

### Statistical analysis

Each experiment involving tissue culture was performed in triplicates. All analyses were performed using the SPSS 13.0 software. Results are expressed as mean ± SD. The one-way analysis of variance (ANOVA) was used to compare the difference between treatment groups. Differences were considered significant if the p value is less than 0.05.

## Results

### Growth inhibitory effect of rapamycin on lung cancer cells

**Figure 1:**
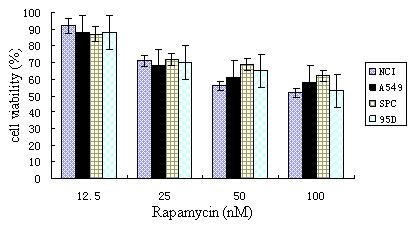
**Rapamycin exerts growth inhibitory effects in four lung cancer cell lines in a dose-dependent fashion**. Cells were treated with increasing levels of rapamycin for 24 hours before cell viability was examined by MTT assay. Control group received treatment of DMSO solution of the same volume and concentration used to dissolve rapamycin.

We first set out to examine whether and at what levels rapamycin inhibits the growth of four different lung cancer cell lines (NCI-H446, A549, SPC-A-1 and 95D). As shown in Figure 1, rapamycin treatment exerted modest inhibitory effect on lung cancer cell proliferation in a dose-dependent manner in all cell lines tested. In addition, the effect of rapamycin seems to level off with its increasing concentration, achieving about 30 - 40% reduction in cell proliferation at 100 nM vs. ~ 10% reduction at 12.5 nM. Finally, the inhibitory effect and its saturating trend towards higher doses of rapamycin are the same for all four cancer cell lines, suggesting rapamycin may act on some targets/pathways common in all of them.

### Growth inhibitory effect of rapamycin with docetaxel on lung cancer cells

**Figure 2:**
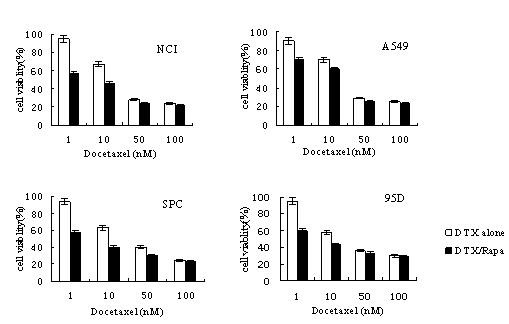
**Rapamycin administered at 20 nM was able to potentiate the growth inhibitory effect of docetaxel in four lung cancer cells**.

Next we checked the effect of rapamycin on docetaxel-induced growth inhibition in lung cancer cells. It was found that 20 nM rapamycin can potentiate the growth inhibition activity of docetaxel in all four cancer cell lines (Figure 2). This enhancing effect of rapamycin is especially pronounced at low docetaxel concentration (1 nM), having led to an additional 20 - 40% of reduction in cell growth. Although rapamycin does not change the maximum level of cell growth inhibition elicited by docetaxel (e.g., at 100 nM), the co-treatment of rapamycin with docetaxel effectively lowered the EC50 (concentration needed to achieve 50% of maximal effect) of the latter.

### Rapamycin induces apoptosis in synergy with docetaxel

**Figure 3:**
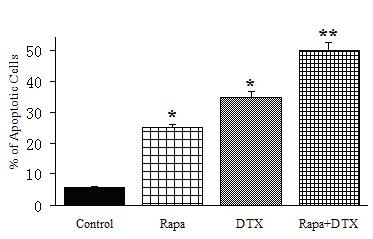
**Rapamycin enhances the apoptosis effect of docetaxel in lung cancer cells**. *P < 0.05, significantly different from untreated control; **P < 0.05, significantly different from either rapamycin or docetaxel monotherapy.

To further investigate whether the enhancing effect that rapamycin showed in docetaxel-co-treated cancer cells is associated with an increased level of apoptosis, we performed flow cytomety analysis using Annexin V/propidium iodide-stained cells. As shown in Figure 3, rapamycin enhances the effects of docetaxel in promoting cancer cell death. Discounting the basal apoptosis level as shown in the control sample, the level of apoptosis in the Rapa+DTX group is close to the sum of those in the two monotreaments using either compound alone. These findings indicate that rapamycin may further enhance the efficacy of docetaxel by inducing a higher degree of apoptosis.

### Combination treatment of rapamycin with docetaxel decreases the expression of Survivin

**Figure 4:**
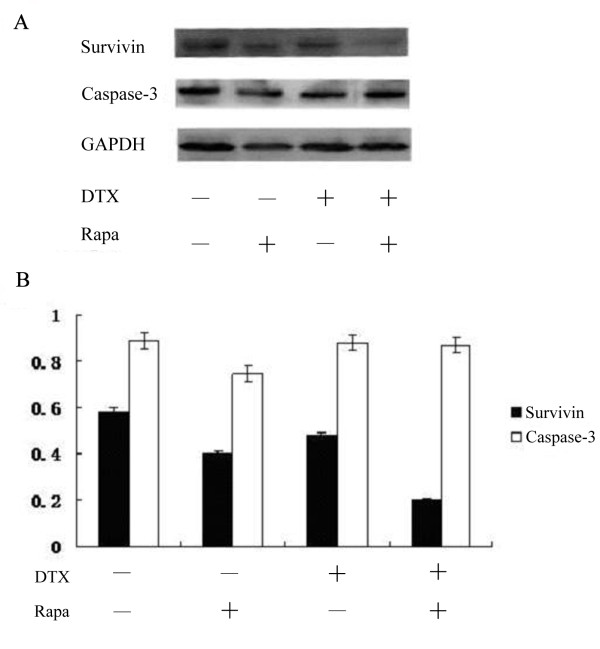
**Rapamycin and docetaxel decrease the level of Survivin expression while the expression of caspase-3 is unaffected**. (A) The presence of various proteins was detected by Western blot. (B) The relative level of Survivin and caspase-3 expression to GAPDH is shown in bar graph.

As we wondered whether the enhancing effect of rapamycin might come from its ability to block cellular pathways that can counteract the cytotoxic activity of docetaxel, the effect of rapamycin on the expression of Survivin was next examined. Treatment of 95D cells with either rapamycin or docetaxel alone resulted in moderate but significant reduction on the level of Survivin expression compared with that of the untreated cells. Moreover, the co-treatment resulted in an even bigger reduction in the Survivin protein level than those of the two single drug treatments added together (Figure 4). In contrast, the expression of a key marker in the apoptotic pathway, caspase-3, is largely unaffected by these treatments.

### Combination treatment of rapamycin with docetaxel decreases the phosphorylation level of ERK1/2 in 95D cell lines

**Figure 5:**
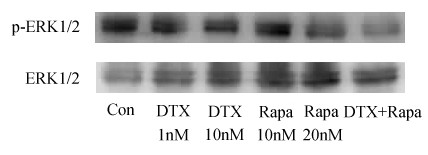
**Combination treatment of rapamycin and docetaxel decreases phosphorylation of ERK in 95D cell lines**. 95D cells were treated with 1 nM and 10 nM docetaxel alone, 10 nM and 20 nM rapamycin alone and a combination with 10 nM docetaxel and 20 nM rapamycin for 24 hr. After incubation, levels of ERK1/2 and p-ERK1/2 (phosphorylated Tyr204) were examined. Con: control, Rapa: rapamycin, DTX: docetaxel.

To further clarify the cell growth inhibitory mechanism of rapamycin with docetaxel, we examined the changes in the expression levels of the enzymes involved in cell growth signal transduction pathways. 95D cells were exposed to rapamycin (10 nM, 20 nM) and docetaxel (1 nM, 10 nM) alone or in combination (Rapa 20 nM+ DTX 10 nM). After 24 hr of incubation, the expression and the phosphorylation levels of ERK1/2 were examined. As presented in Figure 5, a 24-hr exposure to rapamycin or docetaxel alone did not significantly alter the level of expression or phosphorylation of ERK1/2, whereas cells treated with the combination of rapamycin with docetaxel exhibited a marked reduction in the phosphorylation levels of ERK1/2. This suggests that there may exist positive interactions between rapamycin and docetaxel in the suppression of ERK1/2 pathway in 95D cells.

## Discussion

The prognosis for inoperable or recurrent lung cancer patients has not been much improved despite the advent of new chemotherapeutic agents. Although early stage lung cancer is potentially curable, most lung cancer patients were already at advanced stages when diagnosed. Moreover, most advanced lung cancer patients have a history of smoking thus suffer concurrent complications in both cardiovascular and pulmonary systems, rendering aggressive surgery and multimodality therapy unfeasible.

Docetaxel is a common second-line therapeutic agent used for advanced NSCLC. In several randomized clinical tries, combination cytotoxic chemotherapy regimens for second-line therapy of advanced NSCLC failed to establish patient survival benefit, although there was report of higher cytotoxic effect[23]. It has been thought that the clinical benefit of present second-line therapies for advanced NSCLC has reached its peak. More recently, combinations of molecularly targeted agents with standard chemotherapy are being investigated clinically with the hope to surpass the current therapeutic threshold of second-line therapies[24].

Activation of PI3K-Akt-mTOR pathway has been detected in many types of tumors including lung cancer, which is considered to be important for the survival, proliferation, angiogenesis and resistance of cancer cells to chemotherapy[25]. Consequently, this pathway has been regarded as an attractive target of molecular targeting therapy. Indeed, rapamycin treatment has shown some promising antitumor effect in tissue culture systems[19]. However, as evidenced in clinical phase studies, rapamycin analogue monotherapy exerted a modest but limited antitumor effect[26,27]. In order to achieve a greater therapeutic benefit, several combination therapies of rapamycin and other cytotoxic or molecular targeting agents have been under clinical study. Encouragingly, rapamycin has clearly shown either synergistic or additive effects in these treatments[28-30]. In the present study, rapamycin treatment alone exerted modest inhibition on cell proliferation of several lung cancer cell lines in a dose-dependent manner. However, when applied together, the proliferation inhibition effect of docetaxel was significantly potentiated by rapamycin. This observation is in line with previous reports that regarded the mTOR pathway as a promising target of therapy in the treatment of other solid tumors refractory to conventional chemotherapies[31,32].

Apoptosis, induced by chemotherapy, radiation and cytokines, seems to be the main mechanism to kill tumor cells. We suspected that the rapamycin may also enhance the apoptosis-inducing effect of docetaxel in cancer cells. We used flow cytometry analysis to show that rapamycin and docetaxel combination indeed induced higher degree of apoptosis in lung cancer cell lines than that by either compound alone. This led us to further ponder upon the potential downstream effectors of rapamycin and docetaxel-induced signaling pathways in lung cancer cell lines. As a first step, we examined the expression and phosphorylation levels of some proteins known to be involved in cell proliferation and apoptosis. Interestingly, Survivin and ERK1/2 were found to be down-regulated in expression and phosphorylation, respectively, especially by the combination treatment of rapamycin and docetaxel. In comparison, the expression of caspase-3, an apoptosis effector downstream of mitochondrial cytochrome c release, was found to be unaffected.

Survivin is a member of the inhibitor of apoptosis proteins (IAP) family that is typically absent in most normal adult differentiated tissues. However, its mRNA and protein are found in abundance in fetal tissue, most transformed cell lines and cancers. Survivin suppresses apoptosis and promotes angiogenesis, proliferation and metastasis in cancer cells[33-37]. Survivin can block apoptosis by inhibiting terminal apoptotic effectors caspase-3 and caspase-7, and by suppressing both the proteolytic activation and the activity of caspase-9 in the context of Survivin-IAP complexes[38-40]. Clinically, increased expression of Survivin is often associated with elevated resistance of cancer cells to apoptotic stimuli during chemotherapy and is negatively correlated with response to proapoptotic drugs and/or radiotherapy in patients with bladder cancer, breast cancer, lymphoma and multiple myeloma[41-46]. Furthermore, overexpression of Survivin is a prognostic biomarker for decreased patient survival in multiple cancers, e.g., breast cancer, colorectal and gastric carcinomas, neuroblastoma and NSCLC. All these findings on Survivin indicate that it could be an attractive cancer target. In this study, we were intrigued to find that co-treatment with rapamycin and docetaxel significantly down-regulates the expression of Survivin, as shown in Figure 4. Although the underlying mechanism for this down-regulation is currently unclear, our finding is consistent with a previous report that found rapamycin reduced IGF-induced Survivin expression in prostate cancer cells[47]. Similarly, Vaira *et al. *also reported that treatment of rapamycin with taxol at suboptimal concentration resulted in a bigger reduction in Survivin expression than that by either treatment alone[47]. It is possible that when co-treatment of rapamycin and docetaxel synergistically reduced Survivin level beyond the threshold for its antiapoptotic activity in cancer cells, the cytotoxic effect of docetaxel becomes more effective in cancer treatment. In addition, our result suggests that Survivin is essentially involved in lung cancer maintenance and progression rather than initiation, which is in agreement with the prevailing hypothesis. Finally, because Survivin is selectively expressed at the G2/M phase of the cell cycle and is a known mitotic regulator of microtubule assembly, the target of action by docetaxel, it is tempting to speculate an antagonistic interplay between Survivin and docetaxel[48,49]. Interestingly, recent studies are converging on the notion that inhibition of Survivin in conjunction with docetaxel treatment delivers better cancer-killing effect by reversing the resistance to docetaxel in cancer [50,51].

Activation of the MEK/ERK axis is often associated with cell proliferation and survival[52,53]. Similar to Survivin's role in cancer, the phosphorylation level of ERK1/2 is often found upregulated in cancer cells and inhibitors against MEK are currently in Phase II clinical trials. In our study, we found that while monotherapies with either rapamycin or docetaxel did not significantly affect the phosphorylation level of ERK1/2, the combination of the two led to a considerable reduction in the amount of phosphorylated ERK1/2(Figure 5). This is significant, because ERK1/2 activation was known to counteract the cancer-killing activity of docetaxel in some malignancies such as leukemia and melanoma[54-56]. It follows that if ERK1/2 activation is blocked due to the combined effects of rapamycin and docetaxel-induced events, cancer cells may be more sensitized to proapoptotic chemotherapeutics.

## Conclusion

In conclusion, the present study demonstrates that mTOR inhibition by rapamycin suppresses lung cancer cell growth and sensitizes tumor cells to docetaxel-induced cytotoxicity. The rapamycin-dependent enhancement of cancer-killing effects by docetaxel is associated with down-regulation of Survivin expression. Although the precise mechanism of interactions between rapamycin and docetaxel is not presently clear, their proliferation inhibitory and apoptosis-inducing effects may be exerted through down-regulating Survivin expression, either directly or indirectly. Our results suggest that a therapeutic strategy combining specific inhibitor of mTOR with cytotoxic agents may be a promising approach to an improved treatment of advanced lung cancer.

## Competing interests

The authors declare that they have no competing interests.

## Authors' contributions

HYN participated in research design, the writing of the paper, the performance of the research and data analysis. JHW participated in the performance of the research and data analysis. HL participated in the performance of the research. PH participated in research design and data analysis. All authors read and approved the final manuscript.
